# Expression analysis of secreted and cell surface genes of five transformed human cell lines and derivative xenograft tumors

**DOI:** 10.1186/1471-2164-6-55

**Published:** 2005-04-18

**Authors:** Robert A Stull, Roya Tavassoli, Scot Kennedy, Steve Osborn, Rachel Harte, Yan Lu, Cheryl Napier, Arie Abo, Daniel J Chin

**Affiliations:** 1 PPD Discovery, 1505 O'Brien Street, Menlo Park, California 94025, USA; 2 Piedmont Research Center, Morrisville, North Carolina 27560, USA

## Abstract

**Background:**

Since the early stages of tumorigenesis involve adhesion, escape from immune surveillance, vascularization and angiogenesis, we devised a strategy to study the expression profiles of all publicly known and putative secreted and cell surface genes. We designed a custom oligonucleotide microarray containing probes for 3531 secreted and cell surface genes to study 5 diverse human transformed cell lines and their derivative xenograft tumors. The origins of these human cell lines were lung (A549), breast (MDA MB-231), colon (HCT-116), ovarian (SK-OV-3) and prostate (PC3) carcinomas.

**Results:**

Three different analyses were performed: (1) A PCA-based linear discriminant analysis identified a 54 gene profile characteristic of all tumors, (2) Application of MANOVA (Pcorr < .05) to tumor data revealed a larger set of 149 differentially expressed genes. (3) After MANOVA was performed on data from individual tumors, a comparison of differential genes amongst all tumor types revealed 12 common differential genes. Seven of the 12 genes were identified by all three analytical methods. These included late angiogenic, morphogenic and extracellular matrix genes such as *ANGPTL4*, *COL1A1*, *GP2*, *GPR57*, *LAMB3*, *PCDHB9 *and *PTGER3*. The differential expression of *ANGPTL4 *and *COL1A1 *and other genes was confirmed by quantitative PCR.

**Conclusion:**

Overall, a comparison of the three analyses revealed an expression pattern indicative of late angiogenic processes. These results show that a xenograft model using multiple cell lines of diverse tissue origin can identify common tumorigenic cell surface or secreted molecules that may be important biomarker and therapeutic discoveries.

## Background

The process of tumorigenesis has long been recognized to depend upon complex interactions of a tumor with its non-transformed tissue environment [1]. Beyond transformation and increased proliferation, many pathways are activated both in the growing tumor and its environment to culminate in an established solid tumor. For example, adhesive pathways are activated to enable transformed cells to aggregate and form a microtumor. Subsequently, microtumors must avoid destruction by the immune system and elicit vasculature formation for continued growth [2,3]. In support of these events, cell-matrix adhesion proteins, cell surface antigens, angiogenic factors and modulatory agents have been found differentially expressed in several experimental models of tumorigenesis [4-6] and in tumor biopsy samples relative to control tissues [7,8]. Experimental models with established tumorigenic human cell lines have compared the gene expression profiles between the cultured parental cells and after implantation into immune-deficient murine hosts [6]. In this study, we examined this problem with a more focused approach with respect to the transcripts as well as a broader survey by examining multiple tumor sources in order to identify differential genes common to multiple solid tumors in a xenograft model of tumorigenesis.

To recapitulate the attachment and growth of a micro- or metastatic tumor, our experimental tumorigenesis model examined human xenograft tumors in nude mice. It is believed that primary or metastatic microtumors about 1 mm^3 ^in size are metastable; they are either (i) resolved by the immune system, (ii) remain in a steady-state with balanced proliferation and apoptosis or (iii) undergo aggressive growth as long as a vasculature is developed to provide nutrients to the growing mass [9]. Since the end-point of the xenograft assay is the formation of a solid tumor, genes supporting vasculogenesis and angiogenesis are likely differentially expressed relative to the parental cell lines that were adapted to culture in vitro. However, the extent of vascularization to support an established tumor will vary according to the tumor type and tissue environment as a result of variable levels of proteases, receptors or regulators of pericyte and/or endothelial migration, proliferation, and differentiation [3,10]. Additionally, some tumors such as early grade astrocytomas can leverage existing normal brain blood vessels without substantial vasculogenesis for subsequent angiogenic sprouting of new vessels from preexisting vessels [11]. Further, vascularization depends upon a tuned interaction in the tissue microenvironment between endothelial cells and pericytes [12,13]. Vascularization of solid tumors may also be heterogeneous with a rapidly growing margin surrounding a hypoxic core following regression of co-opted vessels that supported early tumor growth [10]. Complicating this picture is the potential for Vascular mimicry' where breast tumor derived cells express endothelial markers and may serve as rudimentary channels [14].

Many angiogenesis studies have used cultured primary vascular endothelial cells and shown the significant roles of *VEGF*, *FGF*, *PDGF*, chemokines and cell-matrix adhesion proteins [3,15,16]. These assays for endothelial cell migration include the chorioallantoic membrane [17], matrigel migration assays [18] or 3D-collagen assays [19]. However, the limits of studying the angiogenic process with established endothelial cells in vitro have been recognized. Tumorigenesis involves both heterophilic and homophilic cellular communication and adhesion between not only endothelial cells but also pericytes and smooth muscle cells; hence other cell surface proteins and secreted factors are absent from such assays [3].

A search for tumorigenic genes common to tumors of diverse origin should be as broad as possible and hence should not be limited to a single tumor type or tissue source. In order to find common tumorigenic genes regardless of tissue origin, we chose to study a panel of 5 adenocarcinoma cell lines from breast, colon, and lung, ovarian and prostate tumors. These cell lines reproducibly yield solid tumors in a standard xenograft assay in immuno-compromised mice [20-22]. While there may be individual differences in capillary branching or density between tumor types, the xenograft assay requires vascular development to support solid tumor formation in a relatively avascular subcutaneous site.

Since the early tumorigenic events largely rely upon secreted factors, cell surface receptors or integral membrane proteins, we devised a strategy to employ a custom microarray to focus on the expression of genes chosen on the basis of their cellular localization. Hence, we implemented an experimental microarray strategy with high replication and coverage of all possible secreted and cell surface proteins. Also, focusing on all known and predicted cell surface and secreted genes allowed us to design more intra-chip replicates for improved data reliability. While prioritizing on the 'Function' category of the Gene Ontology [23], the range of 'Biological Processes' covered by the gene selection remained broad. In contrast to early concerns that a sub-selection of genes might result in a systemic bias, relatively small numbers of genes were found to be common to all xenograft tumors due to the robust experimental design and statistical analysis.

## Results

**Figure 1:**
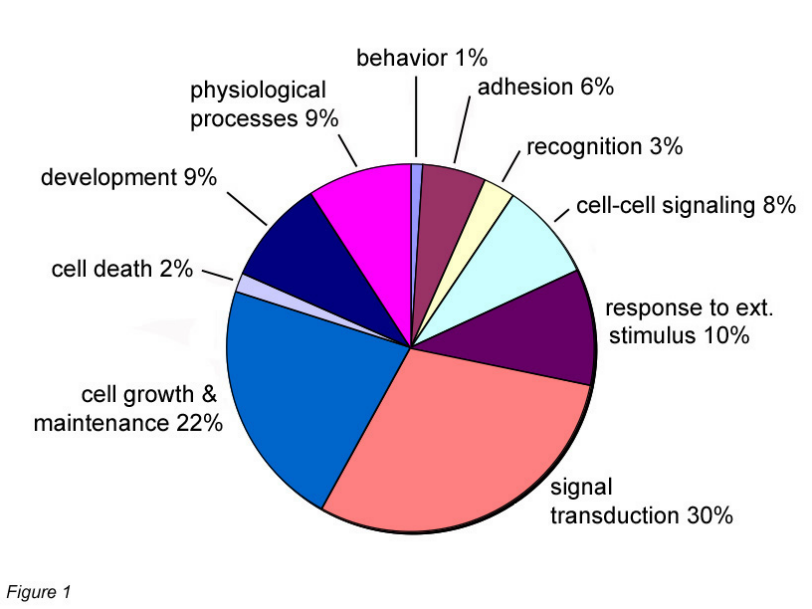
**Gene ontology of custom chip probes**. The ontological classification of 3531 cell surface or secreted genes was extracted from the Gene Ontology at the third level. Genes lacking GO annotations at this level were derived from level 2.

We developed a custom 60-mer oligonucleotide microarray to focus on an ontologically restricted set of secreted and cell surface genes for higher data reliability using a matrix design with intra-chip replicates in addition to replicate chips. Due to the limits of the Gene Ontology classification, multiple strategies had to be used to derive a relatively complete collection of secreted and cell surface genes. For example, some proteins have multiple localization sites on the basis of newer experimental evidence absent from curated databases; e.g. *SORCS3*, *HDGF*. For proteins with multiple cellular localizations, the literature (PubMed, NCBI) was the annotation source for finding other secreted and cell surface proteins. Finally, other putative secreted and transmembrane-encoding genes and exons were analyzed from hypothetical predictions from the UCSC Human Genome. Redundant genes were removed by a combination of blastn/blastp comparisons and manual curation, but many putative membrane-encoding exons of potential proteins were included. A final tally of 3531 genes was composed of 1057 secreted genes, 1338 G-protein coupled receptor (GPCR) genes with the remainder classified as various integral membrane proteins and cell surface proteins. An ontological view of the custom chip's content is shown in Fig. 1. Finally, in consideration of potential global changes of a selected set of genes, numerous positive and negative controls were included in the array design; including genes characteristic of some tumors (e.g. the estrogen receptor for a subset of breast tumors) and many 'housekeeping' transcripts (e.g. β-actin) commonly used to normalize quantitative PCR studies. However, co-hybridizing all samples with a reference cDNA derived from a mixture of 10 human cell lines enabled 'normalization' with respect to feature, chip, and dye for the MANOVA analysis. This strategy minimizes the potential concern for a skewed normalization by a sub-selected gene population or possible differential behavior of the included 'housekeeping' genes in the xenograft tumors.

### Identification of characteristic tumor-specific genes by all tumor data or individual tumor types by multivariate analysis of variation

We performed several multivariate analyses of the microarray data to find characteristic tumorigenic genes. The MAANOVA tools [24] were chosen for their sensitivity and robustness in measuring differential expression versus previous T-test and log-ratio methods using thresholds for induction or suppression. This was particularly important in these studies that used a relatively complex design with on-chip and inter-chip probe replication, multiple tumor samples and tumor types, dye-swap and a common reference RNA sample for all hybridizations. Thus, this strategy helps avoid any systematic bias from using a chip containing probes for only secreted and cell surface genes. We developed a custom database [25] to allow dynamic re-grouping of data to facilitate multiple analytical models such as all tumor data or individual tumor types and their parental cell lines.

Initially, we identified the differentially expressed genes in all tumors relative to all parental cells regardless of tissue origin. Hence compared all the xenograft tumor data to all the parental cell line data without regard to tumor type. Similarly, both the tumor and parental cell line data were compared to the all reference cDNA hybridization data. These data were analyzed by both principal components analysis (PCA) and multivariate analysis of variance (MANOVA).

### Principal components analysis

**Figure 2:**
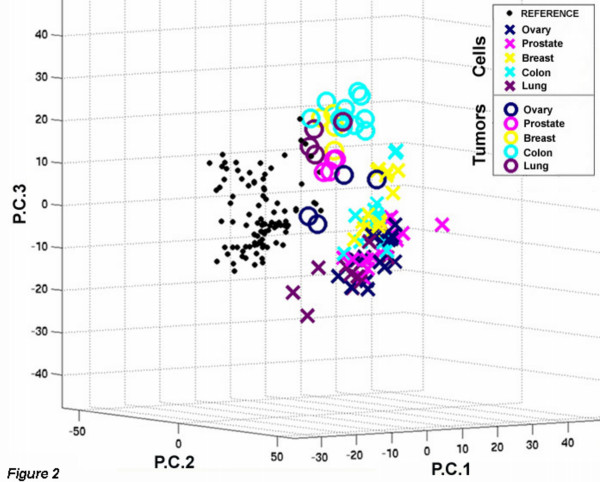
**Principal components analysis of array data**. The mean expression values of all samples from all arrays were analyzed by principal components analysis. The first 3 principal components of the analysis are shown from the best vantage point to show separation of the three classes. Open circles represent the parental cell lines, "X" denotes the various xenograft tumors, and the small solid dots are the reference cDNA sample (derived from the Universal RNA) co-hybridized with all experimental samples. The cell lines corresponding to the various tissue sources of the parental cell lines were: Ovary, SKOV3; Prostate, PC3; Breast, MDA MB-231; Colon, HCT116; and Lung, A549.

To visualize all tumor and parental cell data and assess overall quality, we subjected the entire dataset to principal components analysis. As shown in Fig. 2, a discrete segmentation of the data into 3 major aggregates corresponding to xenografts (circles), parental cell lines ("X's") and the universal reference cDNA (solid dots) can be identified. The third principal component shown by the vertical Y-axis provided the best separation between parental cell data and the xenograft tumor data, Fig. 2.

### Linear discriminant analysis

**Figure 3:**
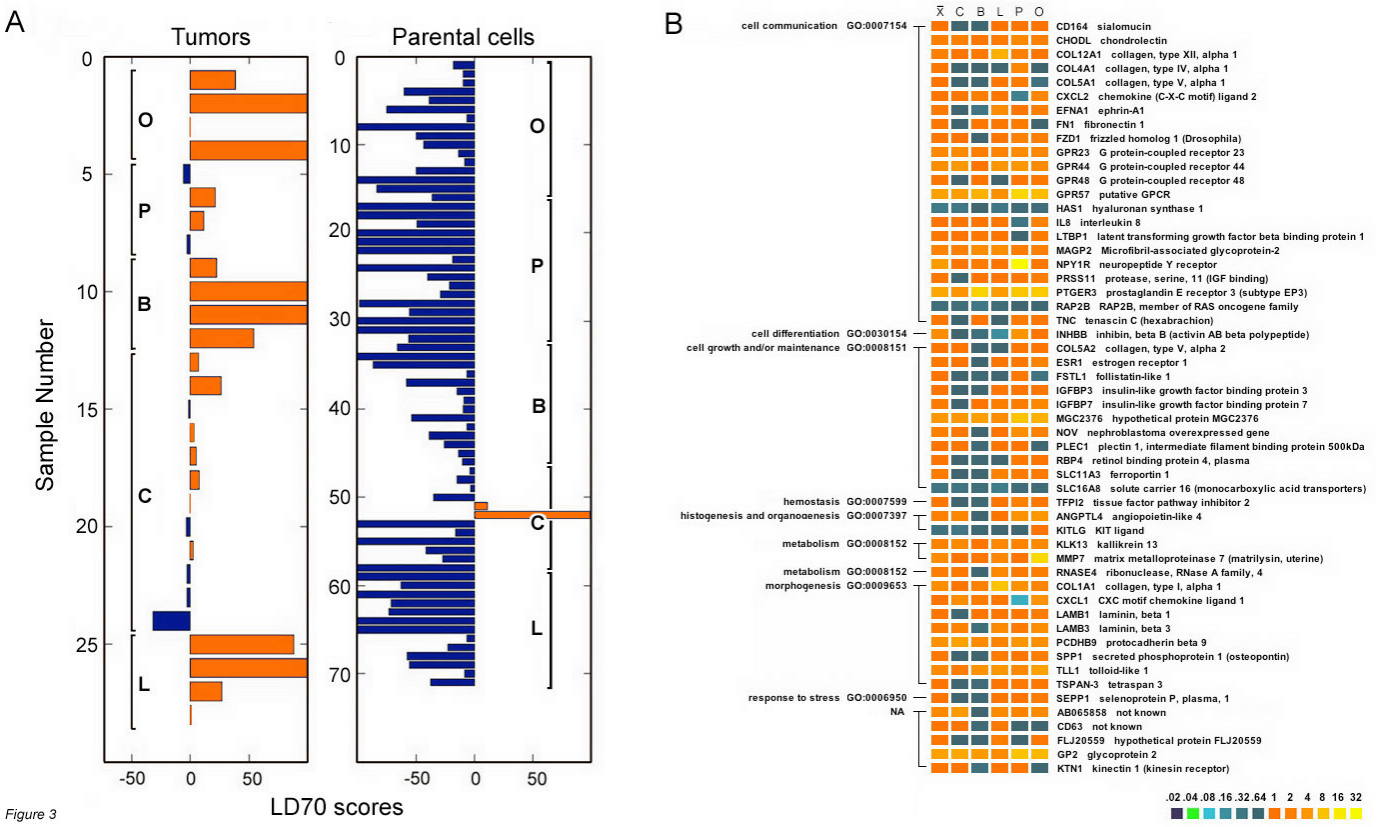
**Genes identified by linear discriminant analysis**. The top 70 PCA coefficients along the third principal component were selected. **Panel A**: Plot of linear discriminant profile of 70 probes that distinguish xenograft tumors from parental cell lines. Positive values in orange indicate "Xenograft tumor" while negative values in blue indicate "Parental Cell line". The y-axis shows either numbered tumor (left) or parental cell (right) samples and the x-axis is an arbitrarily scaled output reflecting the accuracy in assigning a sample as a xenograft tumor or parental cell line. The numbered tumors were grouped according to tissue type as indicated by C for colon (HCT116), B for breast (MDA MB-231), L for lung (A549), P for prostate (PC3) and O for ovary (SKOV-3). **Panel B**: Graphical representation of the LD-p54 genes expression profiles. For genes with multiple probes, the highest value is shown. Classified by a non-redundant filtering of the Gene Ontology biological process terms, the genes are shown with a color scale representing relative fold induction to all parental cell line data. The left-most color column designated by 'X' is the average ratio, while the remaining five columns correspond to Colon (HCT116), Breast (MDA MB-231), Lung (A549), Prostate (PC3) and Ovarian (SKOV-3) carcinoma xenografts respectively.

In order to identify a profile characteristic of xenograft tumors where the combination of multiple genes might be more predictive than any single gene, we performed a linear discriminant analysis. Hence, we iteratively 'trimmed' versions of the third principal component since it had the highest correlation to sample type. The 'trimmed' list of coefficients were tested to determine their accuracy in assigning samples to either the tumor or cell line categories. This analysis retained 70 of the largest coefficients of the third principal component and represents a simple linear discriminant (LD) of 70 probes that corresponds to 54 genes. The profile of 70 probes fairly accurately distinguishes between the two sample types of parental cell lines and xenograft tumors, Fig. 3A. In 'leave-one-out' testing where each of the 99 samples was removed in separate analyses, this method generated a profile that was 79.8% accurate in predicting a xenograft tumor. The same method applied to 1000 label-permuted datasets never exceeded 65% accuracy with a median and minimum accuracy of 49% and 39.3% respectively. This suggests that the gene profile generated by our analysis can distinguish between the xenograft data and the cell line data in a verifiable manner.

### Ontological classification of genes identified by a linear discriminant

The 54-gene profile derived from the linear discriminant (LD-p54) was distributed amongst numerous biological processes using the Gene Ontology classification terms, Table 1. Many genes were classified in multiple biological process categories as a result of their biological complexity; e.g. fibronectin (*FN1*) is classified into 8 biological processes including cell motility, response to stress, cell communication, response to external stimuli, extracellular matrix structural constituent, protein binding and glycosaminoglycan binding. Other genes are involved with cell adhesion or extracellular matrix, cellular growth or the regulation of cellular proliferation, various membrane proteins with known or inferred functions, transporters or channels, and proteases or protease inhibitors. A non-redundant ontological classification of the genes identified by the linear discriminant is shown with a graphical representation of their behavior across all tumor types, Fig 3B. Since the linear discriminant analysis uses a weighted sum, not all of the identified genes behaved consistently across all xenograft tumors; e.g. *CD164 *or *COL4A1*, Fig 3B.

**Table 1:** Gene ontology classification of 54 genes identified by a linear discriminant.

GO	Process	Genes
GO:0006928	cell motility	HAS1 TSPAN-3 FN1 IL8

GO:0006950	response to stress	CXCL2 CXCL1 SEPP1 FN1 SPP1 IL8

GO:0007154	cell communication	MAGP2 LTBP1 PTGER3 COL4A1 COL12A1 IGFBP3 GPR48 CXCL2 PCDHB9 COL5A1 TNC FZD1 CD164 CHODL CXCL1 HAS1 LAMB3 GPR57 EFNA1 FN1 LAMB1 SPP1 GPR23 GPR44 PRSS11 RAP2B INHBB NPY1R ESR1 IL8 KITLG

GO:0007397	histogenesis and organogenesis	KITLG

GO:0007599	hemostasis	TFPI2

GO:0007631	feeding behavior	NPY1R

GO:0008151	cell growth and/or maintenance	FSTL1 NOV IGFBP3 RBP4 MGC2376 CD164 CXCL1 TSPAN-3 SLC11A3 SLC16A8 PLEC1 KTN1 SPP1 COL5A2 PRSS11 INHBB IGFBP7 ESR1 IL8 KITLG

GO:0008152	metabolism	PTGER3 KLK13 HAS1 SEPP1 TLL1 PRSS11 MMP7 INHBB RNASE4 ESR1

GO:0008219	cell death	PTGER3 SPP1

GO:0009605	response to external stimulus	RBP4 CXCL2 CD164 CXCL1 SEPP1 FN1 SPP1 GPR44 INHBB IL8

GO:0009653	morphogenesis	ANGPTL4 COL12A1 PCDHB9 CXCL1 LAMB3 TSPAN-3 SPP1 COL1A1 TLL1 INHBB IL8

GO:0009791	post-embryonic development	INHBB

GO:0016265	death	PTGER3 SPP1

GO:0019058	viral infectious cycle	IL8

GO:0030154	cell differentiation	SPP1 INHBB

GO:0042698	menstrual cycle	INHBB

GO:0046849	bone remodeling	SPP1

GO:0046903	secretion	INHBB

NA	not known	CD63 FLJ20559 GP2 AB065858

A level 3 annotation of the biological process Gene Ontology terms was applied to the list. Due to biological complexity, a gene can occur in more than one category.

### Analysis of variation of all xenograft data

The expression data was also subjected to ANOVA using all xenograft and parental cell line data. In this analysis, the type of tumor or parental line was ignored. This analysis identified 156 probes representing 149 differentially regulated genes at the 99.9% confidence level, Table 2. The range of induction or suppression of this set of genes (ANOVA-p149) was 6-fold induction and 5-fold suppression. Twenty-nine of the 54 genes found by the above linear discriminant analysis were found in the list of 149 ANOVA-qualified probes. An ontological clustering of the ANOVA-p149 genes revealed patterns of proteases and protease inhibitors, cell-matrix adhesion genes, receptors, ion channels, various ligands including chemokines and interleukins, additional angiogenic genes and several genes of unknown function, Tables 3 and 4 show the major ontological groups.

**Table 2:** Differentially expressed genes from three analyses. ANOVA of xenograft data vs parental cell lines found 149 differential genes (designated ' Ap'), Linear discriminant analysis found 54 genes (designated 'LD') and ANOVA of individual xenograft tumors yielded a consensus of 12 genes (designated 'Ai'). For each gene, its presence is denoted by '1' and its absence noted by '0'. The maximum MANOVA Pvalue is reported along with the aggregate ratio (designated by 'R'). For genes with multiple independent probes, the probe reporting the maximum Pvalue is shown. Seven genes common to all three lists are in bold text.

Ap	LD	Ai	Gene	Pval	R
**1**	**1**	**1**	**LAMB3**	0.001	1.9
**1**	**1**	**1**	**ANGPTL4**	0.001	2.1
**1**	**1**	**1**	**COL1A1**	0.001	3.6
**1**	**1**	**1**	**PCDHB9**	0.001	4.0
**1**	**1**	**1**	**GPR57**	0.001	5.7
**1**	**1**	**1**	**GP2**	0.001	5.7
**1**	**1**	**1**	**PTGER3**	0.001	6.4
1	1	0	KITLG	0.001	0.4
1	1	0	RAP2B	0.001	0.4
1	1	0	COL5A1	0.237	1.0
1	1	0	SEPP1	0.054	1.0
1	1	0	CXCL1	0.3	1.1
1	1	0	TNC	0.001	1.3
1	1	0	LTBP1	0.009	1.3
1	1	0	PRSS11	0.001	1.3
1	1	0	FN1	0.008	1.4
1	1	0	FZD1	0.019	1.4
1	1	0	SPP1	1	1.5
1	1	0	IGFBP7	0.001	1.7
1	1	0	RNASE4	0.008	1.9
1	1	0	CHODL	0.003	2.1
1	1	0	NOV	0.003	2.2
1	1	0	COL12A1	0.001	2.2
1	1	0	MAGP2	0.001	2.6
1	1	0	GPR23	0.574	3.0
1	1	0	TLL1	0.001	3.2
1	1	0	GPR44	0.069	3.6
1	1	0	MGC2376	0.001	4.7
1	1	0	NPY1R	0.183	5.2
1	0	1	EMP3	0.004	0.5
1	0	1	HLA-A	0.001	0.6
1	0	1	GNAO1	0.001	2.5
1	0	0	CCR5	0.001	0.2
1	0	0	C20orf52	0.001	0.4
1	0	0	SORCS3	0.001	0.4
1	0	0	PF4	0.005	0.4
1	0	0	SPINK2	0.001	0.4
1	0	0	IGSF6	0.008	0.4
1	0	0	GPR110	0.001	0.5
1	0	0	OR1J5	0.001	0.5
1	0	0	BGLAP	0.001	0.5
1	0	0	GALR2	0.001	0.5
1	0	0	HCN2	0.001	0.5
1	0	0	CD81	0.001	0.5
1	0	0	OGFR	0.001	0.5
1	0	0	GPR6	0.001	0.5
1	0	0	OMP	0.001	0.5
1	0	0	CMA1	0.001	0.5
1	0	0	DKFZP564DO	0.001	0.6
1	0	0	CHRM1	0.001	0.6
1	0	0	PYY	0.001	0.6
1	0	0	FGF19	0.004	0.6
1	0	0	AGTR2	0.047	0.6
1	0	0	SSTR3	0.001	0.6
1	0	0	TMPO	0.001	0.6
1	0	0	TAS2R16	0.003	0.6
1	0	0	ADORA2B	0.003	0.6
1	0	0	GPR10	0.001	0.6
1	0	0	ADCYAP1R1	0.001	0.6
1	0	0	OR1F10	0.001	0.6
1	0	0	HDGF	0.001	0.6
1	0	0	CD151	0.001	0.6
1	0	0	PDAP1	0.001	0.7
1	0	0	A1BG	0.001	0.7
1	0	0	LIPF	0.001	0.7
1	0	0	PBEF	0.001	0.7
1	0	0	ART-4	0.034	0.7
1	0	0	C1QTNF3	0.029	0.7
1	0	0	SLC39A4	0.022	0.7
1	0	0	IFNGR2	0.001	0.8
1	0	0	ENT3	0.001	0.8
1	0	0	SERPINC1	0.001	0.8
1	0	0	NRP1	0.006	0.8
1	0	0	CACNA1H	0.011	0.8
1	0	0	CD44	0.001	0.8
1	0	0	STC2	0.018	0.8
1	0	0	DLK1	0.064	0.8
1	0	0	F2R	0.388	0.8
1	0	0	EMP2	0.001	0.8
1	0	0	HBE1	0.003	0.8
1	0	0	BSG	0.003	0.8
1	0	0	GPR80	0.001	0.8
1	0	0	APOB48R	0.016	0.8
1	0	0	AMELY	0.001	0.8
1	0	0	IL26	0.006	0.8
1	0	0	TRPM5	0.001	0.8
1	0	0	ENSA	0.001	0.8
1	0	0	OR1F1	0.001	0.8
1	0	0	GP3ST	0.001	0.8
1	0	0	BDNF	0.001	0.9
1	0	0	PLXN3	0.005	0.9
1	0	0	APMCF1	0.134	0.9
1	0	0	SCAMP1	0.001	0.9
1	0	0	PALMD	0.001	0.9
1	0	0	MMP8	0.02	0.9
1	0	0	MFAP3	0.004	0.9
1	0	0	SPAG11	0.001	0.9
1	0	0	A2M	0.031	0.9
1	0	0	NET-2	0.092	0.9
1	0	0	CXCL11	0.001	1.0
1	0	0	KLRB1	0.003	1.0
1	0	0	TF	0.988	1.0
1	0	0	COL14A1	0.001	1.0
1	0	0	IL7	0.002	1.1
1	0	0	COL9A1	0.001	1.1
1	0	0	CCR4	0.001	1.1
1	0	0	FPR1	0.034	1.1
1	0	0	FAP	0.001	1.2
1	0	0	OPCML	0.001	1.2
1	0	0	GPR145	0.001	1.2
1	0	0	GFRA3	0.001	1.2
1	0	0	EDN3	0.001	1.2
1	0	0	IL12B	0.043	1.3
1	0	0	CXCR4	0.026	1.3
1	0	0	PCSK5	0.427	1.3
1	0	0	NID2	0.168	1.3
1	0	0	ITGA4	0.73	1.3
1	0	0	KIAA1870	0.016	1.3
1	0	0	FBLN5	0.001	1.4
1	0	0	TRPV2	0.001	1.4
1	0	0	FGF23	0.119	1.4
1	0	0	TEM5	0.001	1.4
1	0	0	CR1	0.008	1.4
1	0	0	GPA33	0.001	1.4
1	0	0	CLCA4	0.001	1.4
1	0	0	TIMP3	0.006	1.4
1	0	0	MMP10	0.001	1.4
1	0	0	FUT8	0.197	1.4
1	0	0	FIBL-6	0.001	1.4
1	0	0	V1RL1	0.001	1.5
1	0	0	EBI2	0.003	1.5
1	0	0	ADAM28	0.001	1.5
1	0	0	GPLD1	0.008	1.5
1	0	0	CP	0.003	1.5
1	0	0	EPHA3	0.003	1.5
1	0	0	KLK11	0.001	1.6
1	0	0	OR7A17	0.001	1.6
1	0	0	IFI27	0.001	1.7
1	0	0	RNASE6	0.003	1.7
1	0	0	SELPLG	0.001	1.7
1	0	0	CST7	0.092	1.7
1	0	0	LEC3	0.001	1.7
1	0	0	TSHR	0.001	2.1
1	0	0	MC2R	0.001	2.1
1	0	0	SV2	0.001	2.1
1	0	0	SERPINA4	0.001	2.1
1	0	0	ANGPT2	0.003	2.2
1	0	0	LOC84664	0.008	2.3
1	0	0	RNASE1	0.001	2.9
0	1	1	HAS1	1	0.3
0	1	0	SLC16A8	1	0.4
0	1	0	CD164	1	1.0
0	1	0	FSTL1	1	1.0
0	1	0	IL8	1	1.0
0	1	0	KTN1	1	1.0
0	1	0	RBP4	1	1.1
0	1	0	COL5A2	1	1.1
0	1	0	TSPAN-3	1	1.1
0	1	0	CD63	1	1.1
0	1	0	IGFBP3	1	1.1
0	1	0	PLEC1	1	1.1
0	1	0	CXCL2	1	1.2
0	1	0	GPR48	1	1.2
0	1	0	FLJ20559	1	1.2
0	1	0	LAMB1	1	1.3
0	1	0	COL4A1	0.994	1.3
0	1	0	TFPI2	1	1.4
0	1	0	ESR1	0.996	1.5
0	1	0	SLC11A3	0.999	1.6
0	1	0	EFNA1	1	1.6
0	1	0	KLK13	1	2.5
0	1	0	AB065858	1	3.1
0	1	0	MMP7	0.987	3.4
0	1	0	INHBB	1	3.5
0	0	1	PI3	1	0.4

**Table 3:** Biological process classification of 175 genes derived from three analyses. The 149 genes derived from the ANOVA analysis of xenograft versus parental cell line data, the 54 genes identified by the linear discriminant analysis and the 12 genes derived from the intersect of ANOVA of individual tumors are shown. Gene Ontology terms were extracted at level 3 for the Unigene gene names. Not shown are genes multiply annotated into additional singular categories or genes absent from the Gene Ontology. Percentages were calculated from a total of 317 classifications into 31 Biological Process terms.

GO	**Process**	**%**	**Genes**
GO:0007154	cell communication	26.8%	HDGF PTGER3 CD44 TAS2R16 GALR2 IGFBP3 COL5A1 OR1F1 SORCS3 FZD1 LAMB3 SELPLG GFRA3 IL26 CXCR4 PDAP1 SSTR3 ENSA CD151 COL9A1 OPCML GPR145 GPR44 EPHA3 TNC GPR80 HAS1 BGLAP EFNA1 EBI2 EDN3 TSHR F2R PRSS11 NRP1 OMP MC2R INHBB OR7A17 IL8 AGTR2 GPR48 CHODL CXCL1 OR1F10 CHRM1 GPR10 GPR57 NID2 GPR6 LAMB1 CCR5 SPP1 ADCYAP1R1 CXCL11 PCSK5 GPR23 RAP2B IFNGR2 IL7 COL12A1 PYY CXCL2 PCDHB9 GPR110 CD164 PBEF KLRB1 FN1 BSG IGSF6 FBLN5 STC2 ANGPT2 ADORA2B PF4 IL12B FPR1 GPLD1 CCR4 NPY1R ESR1 GNAO1 ITGA4 KITLG
GO:0008151	cell growth and/or maintenance	1. 55%	HDGF IGFBP3 SORCS3 TSPAN-3 CXCR4 PDAP1 SSTR3 ENSA TRPV2 SLC39A4 TF EMP3 HBE1 CACNA1H TSHR F2R COL5A2 PRSS11 NRP1 INHBB CLCA4 IGFBP7 IL8 RBP4 MGC2376 CXCL1 CHRM1 A2M CP CD81 CCR5 KTN1 SPP1 SCAMPI OGFR IL7 TRPM5 NOV PYY CD164 PBEF SLC16A8 PLEC1 ANGPT2 HCN2 IL12B EMP2 ESR1 KITLG
GO:0009605	response to external stimulus	12.6%	TAS2R16 OR1F1 IL26 CXCR4 ENSA TRPV2 GPR44 EBI2 EDN3 F2R OMP RNASE6 INHBB OR7A17 IL8 HLA-A RBP4 CXCL1 TIMP3 SEPP1 CD81 CCR5 SPP1 CXCL11 IFNGR2 IL7 CST7 CXCL2 CD164 CR1 KLRB1 FN1 IGSF6 STC2 ADORA2B PF4 IL12B FPR1 CCR4 IFI27
GO:0008152	metabolism	7.9%	PTGER3 LIPF MMP7 EPHA3 KLK11 HAS1 CMA1 F2R MMP10 FAP PRSS11 RNASE6 INHBB MMP8 CHRM1 SEPP1 CD81 PCSK5 RNASE4 KLK13 ADAM28 FIBL-6 TLL1 IL12B ESR1
GO:0009653	morphogenesis	7.9%	BDNF LAMB3 TSPAN-3 GFRA3 CXCR4 CACNA1H BGLAP F2R NRP1 INHBB IL8 AMELY CXCL1 CHRM1 CCR5 SPP1 COL1A1 COL12A1 PCDHB9 FGF19 ANGPT2 TLL1 PF4 IL12B GNAO1
GO:0006950	response to stress	6.0%	IL26 CXCR4 F2R IL8 CXCL1 SEPP1 CCR5 SPP1 CXCL11 IFNGR2 IL7 CXCL2 CR1 KLRB1 FN1 ADORA2B IL12B FPR1 CCR4
GO:0006928	cell motility	3.5%	GALR2 TSPAN-3 HAS1 CACNA1H F2R NRP1 IL8 PYY FN1 FPR1 GNAO1
GO:0008219	cell death	2.5%	PTGER3 CXCR4 SSTR3 EMP3 F2R AGTR2 SPP1 EMP2
GO:0016265	death	2.5%	PTGER3 CXCR4 SSTR3 EMP3 F2R AGTR2 SPP1 EMP2
GO:0030154	cell differentiation	1.9%	BGLAP INHBB SPP1 FGF23 PF4 IL12B
GO:0007397	histogenesis and organogenesis	1.6%	CXCR4 COL9A1 NRP1 IL7 KITLG
GO:0007599	hemostasis	1.3%	SERPINC1 TFPI2 F2R PF4
GO:0000003	reproduction	0.9%	SPAG11 ADCYAP1R1 ADAM28
GO:0007631	feeding behavior	0.9%	GALR2 PYY NPY1R
GO:0009405	pathogenesis	0.9%	CXCR4 EDN3 TSHR
GO:0008015	circulation	0.9%	CACNA1H EDN3 AGTR2
GO:0046849	bone remodeling	0.9%	BGLAP AMELY SPP1
GO:0007586	digestion	0.6%	GALR2 PYY
GO:0019098	reproductive behavior	0.3%	PI3
GO:0030198	extracellular matrix organization and biogenesis	0.3%	COL14A1

**Table 4:** Molecular function classification of 175 genes derived from three analyses. As in Table 3, the gene names from three analyses were annotated according to the Gene Ontology Molecular Function categories. Not shown are genes multiply annotated into additional singular categories or genes absent from the Gene Ontology. Percentages were calculated from a total of 251 gene classifications into 52 Molecular Function terms.

**GO**	**Function**	**%**	**Genes**
GO:0004872	receptor activity	20.3%	PTGER3 CD44 TAS2R16 GALR2 OR1F1 SORCS3 FZD1 GFRA3 CXCR4 APOB48R SSTR3 OPCML TRPV2 GPR145 GPR44 EPHA3 GPA33 TNC GPR80 EBI2 TSHR F2R NRP1 MC2R OR7A17 AGTR2 HLA-A GPR48 OR1F10 CHRM1 GPR10 GPR57 GPR6 CCR5 ADCYAP1R1 GPR23 IFNGR2 OGFR APMCF1 GPR110 CR1 KLRB1 IGSF6 ADORA2B IL12B GPLD1 FPR1 CCR4 NPY1R ESR1 ITGA4
GO:0005102	receptor binding	10.8%	HDGF BDNF SELPLG GFRA3 IL26 ENSA EFNA1 EDN3 F2R INHBB IL8 CXCL1 SPP1 CXCL11 IL7 NOV PYY CXCL2 FGF23 PBEF FBLN5 FGF19 STC2 ANGPT2 PF4 IL12B KITLG
GO:0016787	hydrolase activity	7.6%	RNASE1 LIPF MMP7 KLK11 CMA1 MMP10 FAP PRSS11 RNASE6 MMP8 PCSK5 RAP2B RNASE4 KLK13 ADAM28 FIBL-6 TLL1 GPLD1 GNAO1
GO:0005515	protein binding	7.6%	CD44 TMPO IGFBP3 CXCR4 LTBP1 PRSS11 INHBB IGFBP7 PI3 MGC2376 A2M NID2 CCR5 IFNGR2 SERPINA4 NOV PLEC1 FN1 CCR4
GO:0046872	metal ion binding	6.0%	FSTL1 MMP7 TF CACNA1H BGLAP MMP10 LTBP1 MMP8 CP NID2 PCDHB9 ADAM28 FIBL-6 FBLN5 TLL1
GO:0042277	peptide binding	6.0%	GALR2 SORCS3 CXCR4 SSTR3 OPCML GPR44 F2R MC2R AGTR2 GPR10 CCR5 OGFR FPR1 CCR4 NPY1R
GO:0005201	extracellular matrix structural constituent	4.8%	COL5A1 COL4A1 COL9A1 COL14A1 MAGP2 TFPI2 COL5A2 AMELY COL1A1 COL12A1 FN1 MFAP3
GO:0004857	enzyme inhibitor activity	3.6%	SPINK2 SERPINC1 TFPI2 PI3 AGTR2 A2M TIMP3 SERPINA4 CST7
GO:0015267	channel/pore class transporter activity	2.8%	TRPV2 CACNA1H CLCA4 MGC2376 CHRM1 TRPM5 HCN2
GO:0005539	glycosaminoglycan binding	2.8%	HDGF FSTL1 CD44 COL5A1 SERPINC1 FN1 PF4
GO:0003676	nucleic acid binding	2.4%	TMPO RNASE1 APOB48R RNASE6 RNASE4 ESR1
GO:0000166	nucleotide binding	2.4%	EPHA3 FLJ20559 RAP2B APMCF1 HCN2 GNAO1
GO:0016740	transferase activity	2.4%	EPHA3 HAS1 GP3ST FLJ20559 NRP1 FUT8
GO:0004895	cell adhesion receptor activity	1.6%	CD44 TNC GPLD1 ITGA4
GO:0015075	ion transporter activity	1.6%	TRPV2 SLC39A4 CP SLC16A8
GO:0016301	kinase activity	1.2%	EPHA3 FLJ20559 NRP1
GO:0030246	carbohydrate binding	0.8%	CHODL KLRB1
GO:0005386	carrier activity	0.8%	A2M SLC16A8
GO:0005180	peptide hormone	0.8%	PYY STC2
GO:0008565	protein transporter activity	0.8%	SORCS3 SCAMPI
GO:0008147	structural constituent of bone	0.8%	BGLAP COL1A1
GO:0003800	antiviral response protein activity	0.4%	IFNGR2
GO:0008189	apoptosis inhibitor activity	0.4%	SPP1
GO:0015457	auxiliary transport protein activity	0.4%	ENSA

### Verification of selected genes by quantitative PCR analysis

**Figure 5:**
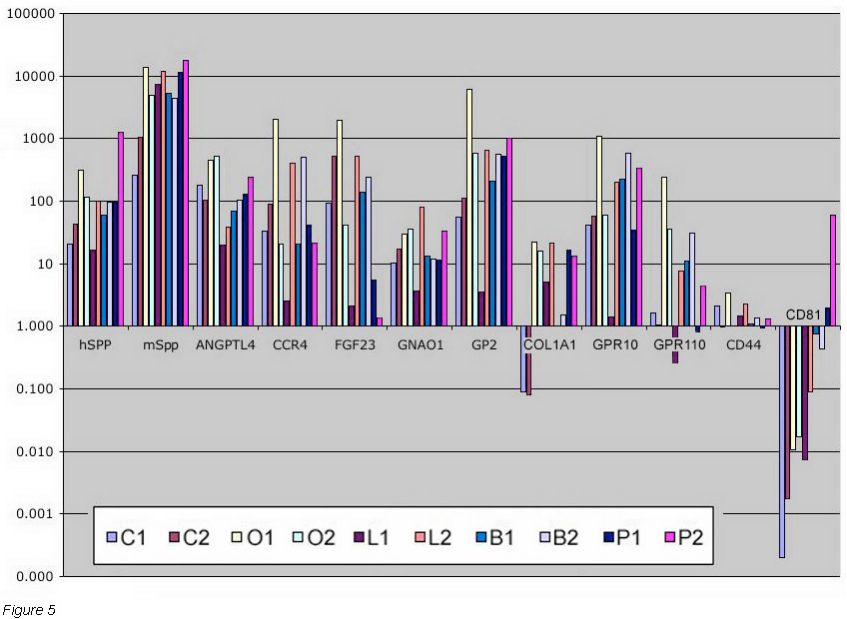
**Quantitative PCR analysis of selected genes**. Two tumors of each tumor type were analyzed by quantitative PCR. The measured fold change relative to cell line was determined. RNA amounts per well being normalized by betaactin signal. In general <2-fold changes are not significant. Hence a call of 1.5 fold down may not actually differ from 1.5 up. Specific tumor types are indicated by the first initial followed by the tumor number: i.e. C1 = colon tumor #1, O1 = ovary tumor #1, L1 = lung tumor #1, B1 = breast tumor #1, P1 = prostate tumor #1.

The differential expression of selected genes was confirmed by quantitative real-time PCR using the same RNA samples subjected to microarray hybridization. The vast majority of the genes tested by PCR validated the array analysis, Fig. 5. In some instances, discrepancies in fold-induction can be explained by methodological differences since the array data were all normalized to the co-hybridized universal-RNA sample, while the PCR data were normalized to a β-actin probe (data not shown). Differential expression of *ANGPTL4*, *GP2*, *GNAO1*, *CCR4*, *FGF23*, *SPP1 *and *COL1A1 *were qualitatively consistent in both the PCR and array analyses. However, two of the down-regulated genes identified by the array analysis, both G-protein coupled receptors, were found by PCR to be elevated, albeit with large variability; *GPR10 *was induced 281-fold SD = 469 and *GPR110 *induced 50-fold SD = 105. Of the two down-regulated genes examined by quantitative PCR, *CD81 *was consistent in both assays, while *CD44 *was measured by PCR as unchanged or minimally induced yet array analysis indicated *CD44 *was suppressed. However, the aggregate 2-fold *CD44 *induction as measured by quantitative PCR is the threshold of what is considered significantly distinguishable from unchanged. Finally, while we did not perform PCR with species-specific probes for every gene present in the ANOVA-p149 list, we were able to confirm differential expression of several human genes from mouse genes such as the osteopontin genes, Fig. 5. While this analysis does not rule out the possibility of partial contamination of the array results by some weak cross-hybridization, to guard against this possibility we carefully designed probes to be species-specific under the stringent hybridization conditions used in this study.

### ANOVA analysis of individual tumor types

To accommodate the possibility that tumor type was an important contributor to differential gene behavior, we performed a third analysis by examining the intersection between the differential genes of each individual tumor type. For this restrictive analysis, we simply examined each tumor type relative to its parental cell line by ANOVA. Approximately 91–312 genes were differentially expressed at 99.9% confidence for each cell line: SKOV-3, 125 differential genes; MDA, 312 differential genes; HCT116, 124 differential genes; A549, 159 differential genes; and PC3, 91 differential genes (data not shown). Twelve genes were found in common amongst these separately analyzed tumor types, *ANGPLT4*, *COL1A1*, epithelial membrane protein 3 (*EMP3*), *GNAO1*, glycoprotein 2 (*GP2*), *GPR57*, *HAS1*, *HLAA*, laminin beta 3 (*LAMB3*), *PCDHB9*, protease inhibitor 3 (*PI3*), and *PTGER3*, Table 2.

### Comparison of multiple analyses

**Figure 6:**
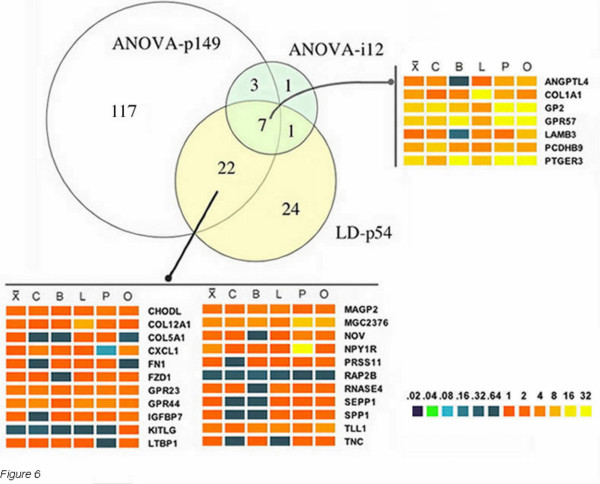
**Overlap of differentially expressed genes identified by three analyses: **ANOVA-p149 = 149 genes derived from the ANOVA analysis of all data, LD-p54 = linear discriminant list of 54 genes from all data, and ANOVA-i12 = twelve genes resulting from a comparison of differentially expressed genes from the ANOVA analysis of individual tumors compared to parental cell lines. An estimate for the statistical significance for the overlap of differentially expressed genes by the 3 analytical methods was estimated by calculating the product of individual probabilities for the results of each analytical method applied to 3531 genes. The null hypothesis in this case is that each method's "call" as to a given gene's differential expression is independent of the call made by the other two methods. Thus if p1, p2, and p3 represent the chance that each method calls a given gene as differentially expressed (easily estimated as number of genes called/ number of total genes), the chance that all three methods do so is simply pAll = p1*p2*p3 = (54/3531)*(149/3531)*(12/3531) = 2.193e-5. Under our null hypothesis, the total number of genes called by all three methods k will follow a binomial distribution with parameters p = pAll, n = 3531 where P(k = L) ~ Bin(pAll, N). Standard calculation techniques allow us to calculate a p-value for this; i.e. p = P(k > = K) – the chance under the null hypothesis we see as much or more overlap than was actually observed. For our data, we thus have p = P(k> = 7) < 1E-6. Thus, if the methods identified random noise as differential expression, they would be very unlikely to produce the overlap observed, thus supporting the statistical significance of the results. The heat maps indicate relative fold-induction or suppression in a linear color-encoded scale shown at the bottom. Mean ratios are indicated by X, C = colon, B = breast, L = lung, P = prostate, O = ovary.

In a typical analysis of multivariate data, a particular method is often chosen as a filter for subsequent analyses. In this study, due to the high statistical reliability imparted by the high replicate probe count (n = 18 to 30) enabled by the custom array design, we chose to compare the results of 3 different approaches to the intact dataset but modeled as either all data or individual tumor types. An estimate of the statistical significance of the overlap in differentially expressed genes common to the three analytical methods gave a Pvalue of < 1 × 10^-6 ^as described in the legend to Fig. 6. As shown in Fig. 6, seven of the twelve differential genes found amongst individual tumor ANOVA analyses were common to the linear discriminant gene profile (LD-p54): *ANGPLT4*, *COL1A1*, *GP2*, *GPR57*, *LAMB3*, *PCDHB9*, and *PTGER3*.

Real-time PCR analysis generally confirmed either induction or suppression in multiple tumor samples but with higher induction ratios; e.g. from Fig. 5, the level of *ANGPTL4 *was measured by PCR as induced 19 to 453 fold with a average fold induction of 185 SD = 170 for 10 tumors (2 of each type). The aggregate induction of *ANGPTL4 *in the array analysis was 2.09 fold (Pcorr < 2e-9). Similarly, *COL1A1 *was measured by PCR as induced in most tumors with an average 9.8-fold (SD = 9.1) versus a 3.64-fold induction found by microarray analysis. Finally, in ovarian and prostate tumors, angiopoietin 2 (*ANGPT2*) measured by PCR was elevated 6-fold (data not shown) versus the 2.2-fold induction found by microarray analysis.

## Discussion

Overall, the pathways represented by the differential genes in xenograft tumors support a model for late angiogenic expression patterns. In light of the collection of xenografts after 28–29 days post-implantation, is not surprising to find patterns of differential gene expression that reflect a portion of the tumorigenic process rather than a preponderance of early transforming events. This premise is largely supported by the abundance of extracellular matrix, cell adhesion and angiopoetic genes common to the three analyses.

Ten of the 12 induced genes identified by the ANOVA of xenografts were either well-characterized functions or biological roles, particularly angiogenesis (ANGPTL4), morphogenesis (*LAMB3*, *COL1A1*, *PCDHB9*, or cellular mobility or communication *(HAS1*, *PTGER3*, *PCDHB9*, and *LAMB3*). The role of extracellular matrix genes in tumor growth has been previously noted [7,8]. Interestingly, five of the extracellular matrix genes from the linear discriminant analysis were collagens (*COL1A1*, *COL4A1*, *COL5A1*, *COL5A2 *and *COL12A1*) and four of these collagens (*COL1A1*, *COL4A1*, *COL5A1*, and *COL5A2*) have been previously found induced in primary renal cell carcinomas (4.8, 5.0, 3.25 and 3.6 fold respectively [26]. *COL1A1 *has also been found induced in most breast carcinomas [27,28], and a subset of ovarian and colon carcinomas [28].

Consistent with an overall pattern of late-stage angiogenesis in xenograft tumors, *ANGPTL4 *was found consistently induced relative to the parental cell lines by all analyses. *ANGPTL4 *originally was described as an induced target of peroxisome proliferator-activated receptor gamma that is involved in glucose homeostasis and differentiation of adipose tissue [29]. Subsequently *ANGPTL4 *was shown to possess angiogenic activity in the chick allochorionic migration assay [30]. More recently, *ANGPTL4 *was shown to bind and inhibit lipoprotein lipase [31], a function consistent with the cachexia induced by tumors, where a reduction of fatty acid incorporation into fat cells serves the energy needs of the tumor rather than the host. *ANGPTL4's *angiogenic action has been reported to be independent of *VEGF *in a renal carcinoma model [30]. Similarly to previous observations of induced angiopoietins in primary renal cell carcinomas (*ANGPT2 *8.18-fold induced and *ANGPTL4 *18–32-fold induced [26], we found both *ANGPTL4 *induction (2.09 fold, Pcorr < 2e-9), and *ANGPT2 *induction (2.23-fold Pcorr < .005).

### Other post-VEGF angiogenic pathways

The role of other elevated angiogenic genes downstream of VEGF bears discussion. The induction of the prostaglandin E receptor 3 (*PTGER3*- 6.4-fold, Pcorr < .001) is of interest since prostaglandins can induce *VEGFA *production [32,33] via a hypoxia-induced pathway [34]. Coincident with these observations, *IGFBP7 *which was differential by ANOVA and linear discriminant analysis, modulates *IGF *mitogenic activity [35]. *IGFBP7 *also stimulates prostacyclin synthesis [36] perhaps to take advantage of our observed 6-fold increased *PTGER3 *expression. Similarly, a human-specific probe for *TEM5*, a marker of tumor endothelial angiogenesis [37], was also found mildly increased (1.37-fold Pcorr < .001) possibly as a result of vasculogenic mimicry [14,38].

**Figure 4:**
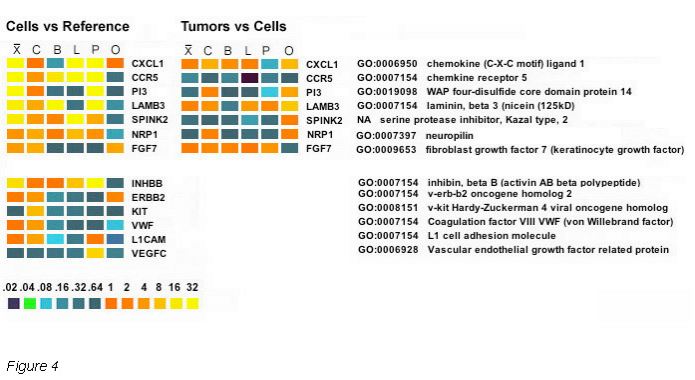
**Comparison of differential expression of genes in parental cells versus reference cDNA synthesized from universal RNA (left) and all tumors versus parental cell lines (right)**. Genes differentially expressed in the parental cells relative to the reference cDNA were analyzed by a 2-way ANOVA (Pcorr < .001). A subset of the differentially expressed genes is shown. The corresponding cognate tumors with differential expression at a 99.9% confidence level by ANOVA analysis of tumors vs parental cell line data are shown. The heat maps indicate relative fold-induction or suppression in a linear color-encoded scale shown at the bottom. Mean ratios are indicated by X, C = colon, B = breast, L = lung, P = prostate, O = ovary.

Other factors such as *FGF *can play an angiogenic role. One *FGF *isoform was found significantly differential in some tumor combinations; *FGF7 *was elevated in colon and prostate xenograft tumors (1.5-fold, Pcorr < 8.7e-6 and 3.7-fold, Pcorr < 7.5e-7) respectively but 2-fold suppressed in ovarian tumors (Pcorr <.006), Fig. 4. *FGF7 *was previously shown to stimulate the growth of endothelial cells of small but not large vessels in the rat cornea [39] and hence supports the notion of vascular remodeling versus vasculogenesis. That differential expression of this gene was found only in some tumor combinations is consistent with the concept that each type of tumor will display individual differences in the balance angiogenic activators and inhibitors, yet the end physiological result, increased tumor vascularization, is the same [3]. Finally, as noted above, genes that help destabilize or remodel vessels such as *ANGPT2 *and *ANGPTL4 *were induced, consistent with an overall pattern of late-stage angiogenesis.

### Linking angiogenic pathways to neuropeptide signaling pathways

Additional support for the late, post-*VEGF *angiogenic pattern of gene expression in xenografts froms from the observed 5-fold induction of *NPY1R *by both ANOVA and linear discriminant analyses. NPY1R has been reported to play a role downstream of *VEGF *in vasoconstriction [40] and capillary sprouting and differentiation [41]. Consistent with the observation of *NPY1R *induction, the potent effect of ligand neuropeptide (*NPY*) upon angiogenesis was shown to yield branching vasodilated structures distinct from those generated by *VEGF *[17]. Similarly, neuropeptide Y has been reported to trigger angiogenesis via the *NPY2 *receptor in ischemic muscle of mice [41]. Interestingly, neuropilin 1 (*NRP1*) which can act as a co-receptor with *VEGFR2 *[42] was found suppressed (1.31-fold, Pcorr < .006) while other *VEGF *receptor levels were not significantly altered. Finally, previous expression profile studies have found *NPY1R *to be substantially induced in many breast, prostate and pancreatic carcinomas [28].

Additionally, two other differential genes involved in neuropeptide signaling were observed: melanocortin-2 receptor (*MC2R*)and *SORCS3*/neurotensin receptor gene. Both *MC2R *and the *SORCS3 *were found differentially expressed by ANOVA. *MC2R *is a GPCR that binds the ACTH peptide while *SORCS3 *is a homolog of the rat sortilin gene with VPS10 domains characteristic to neuropeptide-binding proteins [43-45]. ACTH has been found to increase angiogenesis of cultured endothelial cells in a 3D-collagen assay [19] and other neuropeptides have been implicated in stimulating *VEGF *in prostate cancer cells [46].

## Conclusion

In this study we compared the expression profiles of secreted and cell surface genes from five different tissue sources. Multiple tumors were derived from each parental cell line to examine the potential for tumor heterogeneity arising from the primary isolate, but we found relatively consistent behavior within any tumor group. However, we also found tumor-specific genes for each tumor type while identifying a profile of genes shared amongst all tumor types by multiple analytical approaches. Overall, our results comprise a foundation of commonly regulated tumorigenic genes across tissues such as fundamental angiogenic inducers and regulators. Given the diverse and complex expression behavior of primary human tumors from any single tissue source [27,28], in the future it will be necessary to examine several established lines from many histologically similar primary tumors as well as different tumor types from the same tissue. Similarly, it will be important to compare the effect of orthotopic implantation sites to the subcutaneous injection site in these preliminary studies. To resolve xenograft micro-heterogeneity, microarray analysis of micro-dissected xenograft or primary tumors can be used. Micro-dissection will also allow the assessment of potential vasculogenic mimicry by aggressive tumor cells that can express endothelial genes [38]. Additionally, the xenograft model can be more readily extended to monitor time-dependent expression profile changes in the development of tumors. Such results can be used in combination or as a filter with other biomarker technologies such as tissue arrays [47] or mass spectroscopy [48] to fully characterize clinical specimens for diagnostic or prognostic purposes. By identifying genes known to participate in angiogenesis and tumorigenesis, our work establishes a baseline to evaluate and compare the full spectrum of gene profile changes in xenografts and clinical specimens. Hence, time and tissue-specific gene and protein profiles may be useful for the discovery of both biomarkers and new therapeutic strategies.

## Methods

### Custom array design

A two-stage strategy was employed to design the custom oligonucleotide microarray chip. First, for the known secreted and cell surface proteins, we performed keyword filtering of the gene descriptions and annotations of curated public databases such as SwissProt/Trembl [49], the Gene Ontology tables [23], the UCSC Human Genome assembly (hg13, NCBI Build 31 [50]), the GPCR database [51] and public gene tables from technical supply vendors (Affymetrix, Agilent and Illumina). Some of the keywords used were "secreted", "trans-membrane", "glycosylated" and "olfactory". Redundancies and false positives were removed by manual curation.

In order to accommodate continued optimization of a custom chip design, we chose a chip platform that met several criteria: it must allow rapid changes to the master template even for small production batches, possess relative high density, exhibit strong signal-to-noise properties and have high reproducibility (CV < 10%). Hence, a custom oligonucleotide microarray chip (Agilent, Palo Alto, CA) was designed using the curated collection of secreted and cell surface proteins with human-specific 60-mer probes derived from the 3' 1500 nt region of each mRNA sequence. The custom chip was designed with a matrix of technical probe replicates and multiple probes for some genes; e.g. 2 or 3 probes with 1, 3 or 5 copies each per array represented some genes. All probes were curated by elimination of sequences with unfavorable Tm properties, predicted secondary structure or homo-polymer regions. Finally, Blastn [52] analysis was used to confirm human specificity by comparison to mouse sequences.

### Cell lines and mice

All cell lines (A549, MDA MB-231, HCT-116, SK-OV3, and PC3) were obtained from the ATCC (Manassas, VA). Xenograft tumors were generated from each parental cell line by either implantation of cells or passage of a fragment from a primary tumor (Piedmont Research Center, Morrisville, NC). For the A549, MDA MB-231 and SKOV-3 lines, le-7 cells grown with 10% fetal calf serum in Dulbecco's modified Eagle's medium at 37°C in 5% C0_2 _were implanted subcutaneously into the flank of between 8 and 10 BalbC (Harlan Labs, Indianapolis, IN) mice. Between 50 and 75% of the mice yielded a palpable primary xenograft tumor. For the HCT116 and PC3 xenograft tumors, 1 mm^3 ^tumor fragments between 103–110 mg were excised from a primary xenograft tumor and passaged into secondary mice for the HCT-116 and PC3 xenograft tumors. For PC3 tumors, 8 male mice were implanted with fragments; otherwise recipient mice were female. The number of tumors processed for hybridization were 5 for SK-OV-3, 5 for PC3, 4 for MDA MB-231, 3 for HCT-116 and 5 for A549.

### RNA preparation

For the parental cell lines, total RNA was harvested from 4 x 106 cells using a High Pure RNA isolation kit (Roche Applied Science, Indianapolis, IN) according to manufacturer's instructions. Tumors were excised 22–29 days post-implantation under accredited procedures (Piedmont Research Center, Morrisville, NC), snap-frozen in liquid nitrogen and stored at -80°C until use. Total RNA was prepared from frozen specimens by 24 hr immersion at -80°C in RNAlater-ICE (Ambion, Austin, TX) to 'transition' solid tumors for subsequent homogenization by grinding with a liquid nitrogen-chilled mortar/pestle, followed by resuspension in Trizol (Sigma-Aldrich, E. St. Louis, Mo) and sonication to complete the tissue disruption. Total RNA was extracted using Phase-lock gels (Brinkmann, Westbury, NY), ethanol precipitated, resuspended in RNase-free water, and aliquoted prior to use. Quality control of the total RNA was facilitated by the use of a microcapillary electrophoresis system (Agilent 2100 Bioanlyzer; Agilent Technologies, Palo Alto, CA).

### Experimental design and array hybridization

To identify cell surface genes that are consistently differentially regulated amongst the derivative tumors, multiple tumor specimens and their parental source cell lines were hybridized to the custom chips. All biological specimens were co-hybridized with a reference cDNA synthesized from mRNA that is mixture of 10 human established cell lines (Universal RNA; Stratagene, Carlsbad, CA). For each array, amino-allyl labeled single-stranded cDNA was synthesized from 10 (g of sample total RNA and from 10 ug universal RNA using the Agilent Fluorescent Direct Label Kit according to manufacturer's instructions, except that a dNTP mix containing 5-[3-Aminoallyl]-2'-deoxyuridine 5'-triphosphate (AA-dUTP; Sigma-Aldrich) was used (final concentration: 100 (M dATP, dCTP, dGTP; 50 (M dTTP, AA-dUTP). Amino-allyl labeled cDNA was purified using QIAquick PCR columns (Qiagen, Valencia CA) and coupled to either N-hydroxysuccinimidyl-esterified Cy3 or Cy5 dyes (Cy-Dye mono-functional NHS ester; Amersham, Piscataway NJ). Dye-conjugated cDNAs were purified from free dye using the CyScribe GFX purification kit (Amersham). Targets were hybridized to the microarray for 16 hrs at 60°C using an Agilent In Situ Hybridization Kit per manufacture's instructions, washed 10 min in 6× SSC, 0.005% Triton X-102 at 22°C, 0.1× SSC, 0.005% Triton X-102 for 10 min at 4°C, dried under a stream of nitrogen, and scanned with an Agilent Microarray Scanner. Hybridization signals were extracted with Agilent Feature Extraction Software version 7.1, which yielded the median of all pixel intensities for each feature. Since two identical arrays of 8500 features were printed on each chip, a complete dye-swap comparison could be performed per chip. For example, on the left array, a Cy3-labeled biological specimen was co-hybridized with Cy5-labeled cDNA made from universal RNA. For the cognate dye-swap experiment on the right array, a Cy-5 labeled biological specimen was co-hybridized with Cy3-labeled cDNA made from universal RNA. No tumor samples were mixed with any other tumors.

To enable identification of differentially expressed genes with higher statistical reliability, we performed a matrix of hybridizations. The hybridization matrix follows: for the 5 SK-OV-3, A549 and PC3 tumor specimens, 3 of the tumor samples were hybridized to 2 chips each (hence 4 arrays per tumor sample) while 2 tumor samples were hybridized to a 1 chip each (hence 2 arrays for each of these tumors). For the 4 MDA MB-231 tumor specimens, 3 of the tumor samples were hybridized to 2 chips each and 1 tumor was hybridized to a single chip of 2 arrays. For the 3 HCT-116 tumor specimens, all 3 tumors were hybridized to 2 chips each (4 arrays each). For the parental cell lines, HCT-116 cells were hybridized to 6 chips (12 arrays) while the other cell lines were hybridized to 2 chips each (4 arrays). Since most probes were present minimally in triplicate on each array, whenever a tumor sample was hybridized to 2 chips n = (3*4) = 12 per probe. However, since dye-swap hybridizations were routinely performed, n = 6 for the Cy3 and Cy5 signals respectively.

### Quantitative PCR

Real-time (RT-) PCR analysis of selected RNA transcripts was performed using either a GeneAmp 5700 Sequence Detection System or an ABI PRISM 7900HT Sequence Detection System with SyBr green chemistry (Applied Biosystems, Foster City, CA). The cDNA produced by reverse transcribing the equivalent of 10 ng of total RNA was loaded per RT-PCR reaction. The following primers pairs were used: beta actin (*ACTB*) CCTGGCACCCAGCACAAT, GCCGATCCACACGGAGTACT; Human osteopontin (*HSPP*) AGCAAAATGAAAGAGAACATGAAATG, TTCAACCAATAAACTGAGAAAGAAGC; murine osteopontin (*mSpp*) ATTTTGGGCTCTTAGCTTAGTCTGTT, GGTTACAACGGTGTTTGCATGA; angiopoietin-like 4 (*ANGPTL4*)ATGTGGCCGTTCCCTGC, TCTTCTCTGTCCACAAGTTTCCAG; chemokine (C-C motif) receptor 4 (*CCR4*)ATTCCTGAGCCAGTGTCAGGAG, CTGTCTTTCCACTGTGGGTGTAAG; fibroblast growth factor 23 (*FGF23*)GGCAAAGCCAAAATAGCTCC, CTGCCACATGACGAGGGATAT; G protein, alpha activating activity polypeptide O (GNAO1) CTAGTCTTTGGGAAACGGGTTGT, AAATCCAACACGGCAAAGGA; glycoprotein 2 (*GP2*) GCTTTCCACTCCAATTCACACA, CCTGGCCTTGATTCTGTTAATACC; collagen, type I, alpha 1 (*COL1A1*)TCCCCAGCTGTCTTATGGCT, CAGCACGGAAATTCCTCC; G protein-coupled receptor 10 (*GPR10*)CATGCTCGAGTCATCAGCCA, TTTCACTGCCCCCTTTGTGT; G protein-coupled receptor 110 (*GPR110*)AAGCTCTGGAGGCCGACTG, GGCCTTGTCATCCCGACTC; (*CD44*)TACAGCATCTCTCGGACGGAG, GGTGCTATTGAAAGCCTTGCA; (*CD81*)CCCTAAGTGACCCGGACACTT, CGTTATATACACAGGCGGTGATG. The identity of each amplicon was confirmed by melting curve analysis at the end of the RT-PCR run.

### Array analysis

While the array vendor's feature extraction software 'processed' the hybridization signal to correct for image intensity, background and minor spatial artifacts, chip-to-chip comparisons such as 'reference' versus 'experimental' sample were handled by a custom database [25] built in MySQL [53] with a web interface served by Apache [54]. The database allows the control of experimental design and specification of comparisons and analyses to be performed. Some calculations, like T-Tests and ratios, can be performed in the database or its interface layer, but MATLAB (Mathworks, Natick, MA) was used for ANOVA and principal components analysis (PCA).

For identification of differentially expressed genes, we used the MAANOVA package [55] an implementation of ANOVA for microarray analysis [24]. Array data were loaded into the database and minimally pre-processed for use with this package: where replicate features of the same probe existed in the array design, an arithmetic mean was calculated to yield a single expression level for each probe for each chip. Each tumor or cell line sample was hybridized to 3 separate chips. All signals were Log2 transformed prior to subsequent analyses. These data were used to fit a linear model with factors Gene, Array, Array × Gene, Dye, Dye × Gene, and Sample × Gene. This last attribute is the quantity used for analysis, representing the differential expression of a given gene under a given experimental condition, with the other factors serving to normalize the data. In order to identify differential expression these residuals were analyzed with three statistical tests: a standard ANOVA F-test and two minor variations. A probe had to pass these three tests, generally at 99.9% significance, in order to be called as differentially expressed. A permutation analysis and one-step multiple comparisons correction were applied in conjunction with these tests. It should be noted that since three tests are applied, three P-values result, and when single P-values are listed; the maximum of the three P-values is reported. Finally, since all samples were co-hybridized with cDNAs made from a universal RNA sample, for comparisons of differential gene behavior, approximate 'ratios' were calculated by dividing the paired individual tumor/universal RNA ratio by the paired parental cell/universal RNA ratio.

### Ontology annotation

Unigene Gene names were classified by the consistent terms of the Gene Ontology(tm) consortium [23] and the fatiGO interface to the Gene Ontology [56].

## Authors' contributions

RAS helped design and implement the experimental strategy by developing many protocols, carried out many of the hybridization experiments and analyzed the PCR data. RT carried out the sample preparation, labelings, microarray and PCR experiments. SK performed the statistical analysis and assisted database design. SO designed and built the microarray database and LIMs. RH and YL helped curate, annotate and design the custom microarray chip design. CN carried out the xenograft studies. AA conceived of the experimental design. DJC designed & managed the experimental strategy, helped curate the gene lists and wrote the manuscript with input from co-authors.
